# Psychophysiological stress influences temporal accuracy

**DOI:** 10.1007/s00221-023-06676-9

**Published:** 2023-08-02

**Authors:** Nicola Cellini, Simon Grondin, Franca Stablum, Michela Sarlo, Giovanna Mioni

**Affiliations:** 1 Department of General Psychology, University of Padova, Via Venezia 8, 35131 Padua, Italy; 2 École de Psychologie, Université Laval, 2325 Rue Des Bibliothèques, Québec, QC G1V 0A6 Canada; 3 Department of Communication Sciences, Humanities and International Studies, University of Urbino, Via Saffi 15, 61029 Urbino, Italy

**Keywords:** Autonomic nervous system, Time bisection task, Finger tapping, Heart rate, Skin conductance, Time perception

## Abstract

Distortions of duration perception are often observed in response to highly arousing stimuli, but the exact mechanisms that evoke these variations are still under debate. Here, we investigate the effect of induced physiological arousal on time perception. Thirty-eight university students (22.89 ± 2.5; 28 females) were tested with spontaneous finger-tapping tasks and a time bisection task (with stimuli between 300 and 900 ms). Before the time bisection task, half of the participants (STRESS group) performed a stress-inducing task, i.e., the Paced Auditory Serial Addition Test (PASAT), whereas the other participants (CONTROL group) performed a control task, the Paced Auditory Number Reading Task (PANRAT). The PASAT induced a greater heart rate, but not electrodermal, increase, as well as a more unpleasant and arousing state compared to the PANRAT. Moreover, although the two groups presented a similar performance at the finger-tapping tasks, participants in the STRESS group showed better temporal performance at the time bisection task (i.e., lower constant error) than the controls. These results indicate that psychophysiological stress may alter the subsequent perception of time.

## Introduction

Although humans are able to measure the passage of time accurately in the milliseconds-to-seconds range, our sense of time can be altered and distorted. Indeed, there is a dynamic relationship between subjective and objective perception of time, such that time may be perceived as lasting shorter or longer than the standard unit depending on the context. More specifically, variations in arousal levels are reported to have a major impact on time perception (Wearden 2016). The mechanism by which arousal is thought to influence perceived time has been formulated in the framework of the Scalar Expectancy Theory (SET; Gibbon et al. 1984). According to this model, the raw material for time representation comes from a clock stage consisting of a pacemaker that emits pulses at a given rate, a switch controlling how the pulses are gated, and an accumulator in which the number of pulses is stored during the event(s) being timed. Temporal judgments also depend on memory and decision stages. The model does indeed posit that the rate of the pacemaker pulses is influenced by arousal (Meck 1983; Penton-Voak et al. 1996; Treisman et al. 1990). This way, an increase in arousal level might induce a speeding-up of the internal clock system, leading to a duration overestimation (Buhusi and Meck 2002, 2005; Droit-Volet and Meck 2007; Gil and Droit-Volet 2011, 2012; Grondin et al. 2014, 2015).

Another theory that accounts for physiological arousal on time perception is the interoceptive salience model by Craig (2009), which proposes the idea that temporal processing is influenced by psychophysiological activity associated with emotion. Based on this model, we may expect that stressful experiences able to produce psychophysiological responses may also influence temporal estimation. A previous study provided some support for this model by demonstrating that physiological activation (i.e., skin conductance increase) in response to negative stimuli is associated with time dilation (Mella et al. 2011).

One problem in the timing literature is the definition of arousal, which is still widely discussed as different facets of arousal have been considered in prior studies. The majority of the studies used emotional stimuli to manipulate the arousal level (i.e., *emotional arousal*; Pfaff 2006). Within this context, emotional stimuli generating high arousal led to a greater overestimation of time, compared to emotional pictures generating less arousal (Droit-Volet and Meck 2007; Lake et al. 2016). Specifically, facial expressions conveying anger, fear, happiness, and sadness tend to lead to an overestimation of time compared to neutral stimuli (Droit-Volet and Gil 2009; Gil and Droit-Volet 2011; Lee et al. 2011). However, facial expressions of shame tend to result in an underestimation of time compared to neutral facial expressions (Droit-Volet and Meck 2007; Droit-Volet and Gil 2009; Mioni et al. 2016a). The influence of disgust on time perception is more mixed. While some studies indicate that viewing facial expressions of disgust does not lead to time distortions (Gil and Droit-Volet 2011), other studies suggest that viewing disgusting images, such as body mutilations, can result in a perception of longer duration (Angrilli et al. 1997; Grondin et al. 2014; Mioni et al. 2021). Furthermore, perceiving images of disgusting food can lead to a perception of shorter duration (Droit-Volet and Gil 2009; Mioni et al. 2021).

More germane to the present study are the studies testing the effect of both psychological (i.e., subjective) and physiological arousal on time perception. The latter refers to a body response denoting a state of readiness and involves the activation of the autonomic nervous system (Wearden and Penton-Voak 1995). Some studies manipulated physiological arousal, assessed using heart rate (HR) and/or electrodermal activity (EDA), by increasing physical activities pre- and post-timing tasks (i.e., cycling; Lambourne 2012 used a time generalization task; Vercruyssen et al. 1989 used a time estimation task) or by increasing muscle tension and breath-holding exercise during the timing task (Schwarz et al. 2013). These studies provided no clear-cut results. Indeed, Lambourne (2012) and Schwarz et al. (2013) reported temporal overestimation while Vercruyssen et al. (1989) reported temporal underestimation during physical exercise. Moreover, no clear effect of HR activity on perceived duration was reported by Lambourne (2012) and Vercruyssen et al. (1989), whereas Schwarz et al. (2013) suggested that HR itself has no relevant impact on time perception. Mella et al. (2011) used a time generalization task in which arousal was manipulated using negative and neutral sounds (low vs. high level of arousal) and assessed using skin conductance. The authors reported a lengthening effect of negative emotion on time perception. However, the authors suggested that the link between autonomic arousal and subjective duration is not as direct as was predicted. Dormal et al. (2018) reported verbal temporal overestimation after HR manipulation irrespective of the condition (cycling, relaxation, and crossword), implying that changes in physiological arousal alone cannot explain the temporal bias observed. Similarly, Piovesan et al. (2018) showed that increased skin conductance activity due to task-irrelevant pain stimulation did not affect verbal time estimation. In a very interesting study, Ogden et al. (2019) manipulated the level of parasympathetic activity using normal and paced-breathing exercises before a time estimation task of highly arousing unpleasant and neutral pictures. They showed that, regardless of the pictures’ valence, higher parasympathetic activity induced by the paced breathing was associated with lower temporal accuracy. Lastly, van Hedger et al. (2017) tested the change in the ability to accurately reproduce the duration of highly unpleasant, neutral, and pleasant pictures before and after a social stress task. They observed a temporal dilation after the social stress task for negative and positive pictures.

All these studies are showing a complex relationship between temporal processing and psychological and physiological arousal. In this context, the present study aims to further investigate the effect of psychophysiological arousal on time processing by inducing stress *before* a temporal task. Specifically, we investigated whether increasing individuals’ subjective and physiological arousal (as indexed by skin conductance level and heart rate reactivity) can modulate temporal performance. To manipulate physiological arousal, we engaged participants with a stressful task namely Paced Auditory Serial Addition Test (PASAT; Gronwall 1977), or with a control task namely Paced Auditory Number Reading Task (PANRAT, Tanosoto et al. 2015) before performing a time bisection task, using a between-subject design. If psychophysiological arousal acts at the level of the internal clock (Craig 2009; Gibbon et al. 1984) we should observe temporal modulation in participants included in the stressful condition.

## Materials and methods

### Participants

Thirty-height University students without acute or chronic disease participated in the study (mean age = 22.89 years; SD = 2.5; 10 males and 28 females). This sample size was based on a power analysis which revealed that 36 participants are needed to detect a within-between interaction of interest with an effect size as small as *f* = 0.25 (i.e., Cohen’s *d* = 0.5, a medium effect size) and a statistical power of (1–*β*) = 0.95 (given *α* = 0.05 and a correlation between repeated measures of *r* = 0.5). Before the experimental session, participants were randomly assigned to the Stress (*n* = 19; 15F), or Control (*n* = 19; 13F) group (see Table 1 for the descriptive data of the sample). The study procedure was approved by the local ethical committee, and it was conducted in accordance with the Declaration of Helsinki. All participants provided their written informed consent to participate in the study.

**Table 1:** Summary of results of demographic, self-report questionnaires, and physiological data at rest

	Control		Stress		*t*	*p*	ES	All sample	
	*N* = 19		*N* = 19					*N* = 38	
	Mean	SD	Mean	SD				Mean	SD
Age	22.63	1.86	23.16	3.01	0.659	0.520	0.211	22.89	2.48
MEQr	13.74	3.02	15.67	3.65	1.758	0.087	0.578	14.68	3.43
STAI-Y1	32.35	5.98	33.84	7.61	0.647	0.522	0.216	33.14	6.83
SSS	1.58	0.61	1.90	0.66	129.5*	0.149	0.243	1.74	0.64
BFI traits									
Extraversion	3.11	0.79	3.12	0.74	0.061	0.952	0.020	3.11	0.75
Agreeableness	3.76	0.69	3.43	0.66	− 1.525	0.136	− 0.495	3.55	0.69
Conscientiousness	3.64	0.63	3.69	0.73	0.159	0.875	0.051	3.66	0.67
Neuroticism	3.30	0.76	3.23	0.63	− 0.319	0.752	− 0.103	3.27	0.69
Openness to experience	3.55	0.58	3.73	0.76	0.904	0.372	0.293	3.62	0.61
HR (bpm)	78.51	8.98	79.09	10.06	− 0.420	0.627	− 0.136	79.16	9.43
EDA global mean (µS)	5.28	3.92	2.88	1.44	99.0*	0.017	0.452	4.08	3.16
SCr (number)	172.53	56.07	164.11	88.22	1.21	0.233	0.393	168.32	73.03
SCr Amplitude (µS)	0.11	0.09	0.08	0.05	162.0*	0.599	0.102	0.09	0.09

*MEQr* Morningness–Eveningness questionnaire reduced version, *IRI* mean inter-response interval, *STAI-Y1* State‐Trait Anxiety Inventory, form Y, *SSS* Stanford sleepiness scale, *BFI traits* Big Five traits (neuroticism, extraversion, openness to experience, agreeableness, and conscientiousness), *ES* Effect Size, *EDA* electrodermal activity, *SCr* Skin conductance response

*Maan-Whitney *U* test

### Procedure

Upon arrival in the laboratory, electrodes were attached to the participants. After that, participants filled out questionnaires investigating personality traits, state sleepiness and anxiety, and circadian preferences (see below for the description of the questionnaires). Then, after recording a 5 min resting-state physiological baseline in a sitting position, participants performed a finger tapping task (Spontaneous Tempo task), followed by either the Paced Auditory Serial Addition Test (PASAT; Gronwall 1977), used as a stressed task, or the Paced Auditory Number Reading Task (PANRAT, Tanosoto et al. 2015), used as control task, and by a time bisection task. For all tasks, stimulus presentation and data collection were controlled using E-Prime 2.2 (Psychology software tools, Inc., Pittsburgh, PA).

### Measures of time perception

#### Spontaneous tempo task

The spontaneous tempo task is a finger-tapping task in which participants were required to tap with their preferred index finger on the space bar, as regularly as possible at the pace they preferred (free tempo; Mioni et al. 2016b). Both the beginning and the end of the task were marked by a visual stimulus (white cross presented at the center of the computer screen). The participants began to tap when they first saw the cross and continued until the cross disappeared, which was after 45 inter-tap intervals.

#### Time bisection task

The time bisection task was divided into two phases (see Cellini et al. 2015). In the learning phase, participants were required to memorize the two standard intervals presented 10 times (standard short = 300 ms; standard long = 900 ms). The stimulus used was a grey circle presented at the center of the computer screen. During the experimental phase, participants were required to judge different temporal intervals and decide if they were closer to the “standard short” or “standard long” interval. The participants were asked to respond with their left and right index fingers and response keys were counterbalanced between participants. After the response, there was a 1000 ms inter-trial interval. Participants performed 4 blocks; in each block, 7 different comparison intervals were presented, including the two standards (300, 400, 500, 600, 700, 800, and 900 ms). Within each block, each interval was presented 10 times in random order.

### PASAT

**Fig. 1:**
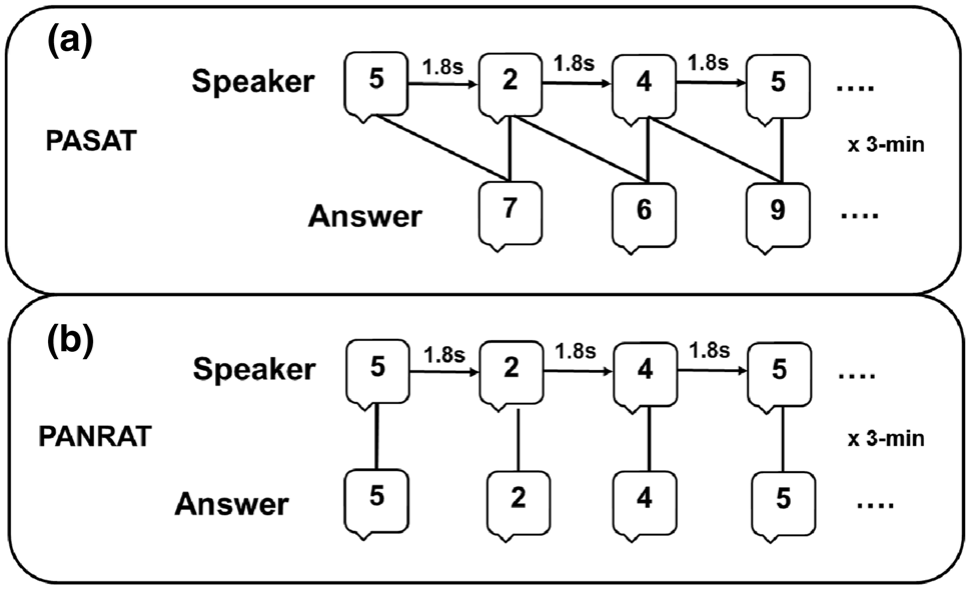
**a** Schematic representation of the Paced Auditory Serial Addition Test (PASAT) used in the current study. A series of digits were continuously presented via speakers every 1.8 s for 3 min. Participants were required to continuously add each new digit to the one immediately before and verbally providing the answer. **b** Schematic representation of the Paced Auditory Number Repetition Test (PANRAT) used in the current study. A series of digits were continuously presented via speakers every 1.8 s for 3 min. Participants were required to repeat aloud each digit they heard

The PASAT is a measure of cognitive function that specifically assesses auditory information processing speed and flexibility, as well as calculation ability. Here the PASAT was used to induce temporary subjective and physiological stress (Tanosoto et al. 2012, 2015) before performing the time bisection task. In the current version of the task (Fig. 1a), a list of 61 single digits was presented via speakers every 1800 ms for 3 min; participants were required to add each new digit to the one immediately before it. In the control version (PANRAT, Fig. 1b), participants were required to repeat each digit aloud without doing any addition. Before the beginning of the task, participants performed a 30 s practice trial to acclimatize to the task. At the end of each task, participants rated their subjective valence (i.e., state of pleasantness evoked by performing the task) and arousal (i.e., state of activation evoked by performing the task) using two 9-point graphic scales (from 1 to 9) of the computerized version of the Self-Assessment Manikin (SAM; Lang et al. 1999). Both the PASAT and the PANRAT were preceded by a 1-min resting state, as a physiological baseline for the task.

### Self-report questionnaires

Since personality and circadian preferences seem to affect time perception (Bisson and Grondin 2020; Hammerschmidt and Wöllner 2023; Hornik et al. 2010; Momi et al. 2023; Rammsayer 1997), we used the 44-item version of the Big Five Inventory (BFI; John et al. 1991), translated into Italian (Ubbiali et al. 2013), to assess personality in our sample. The BFI organizes personality into five broad traits (Neuroticism, Extraversion, Openness to Experience, Agreeableness, and Conscientiousness; John and Srivastava 1999; McCrae and Costa 1999). Higher trait scores indicate higher levels of the respective personality traits. Circadian preferences were assessed using the Morningness–Eveningness questionnaire reduced version MEQr (Natale et al. 2006). Finally, to account for potential psychophysiological state-dependent effects, we assessed pre-task sleepiness and anxiety with the Stanford Sleepiness Scale (SSS; Hoddes et al. 1973) and the State-Trait Anxiety Inventory-Y (STAI-Y1; Spielberger 2010), respectively.

### Electrophysiological indices

Electrocardiogram (ECG) was recorded at 512 Hz sampling rate using a modified Lead II Einthoven configuration with spot electrodes. Kubios HRV Analysis Software 2.0 (MATLAB, Kuopio, Finland) was used to automatically detect, examine, and manually edited the R-peaks of the ECG recordings and compute the heart rate (HR). Electrodermal activity (EDA) was measured using two disposable Ag/AgCl electrodes attached to the medial phalanx of the index and middle finger of the left hand. The signal was recorded via a Grass AC/DC strain gage amplifier (Grass Technologies, Astro-Med Inc., West Warwick, RI, USA), then converted with a BIOPAC MP100A A/D and the AcqKnowledge 4.1 (Biopac Systems, USA). EDA pre-processing and parameters computation was conducted with Ledalab (V3.4.9; http://www.ledalab.de), downsampling data to 10 Hz and an 8-point Gaussian smoothing. Artifacts were visually identified and corrected using a spline interpolation. A continuous decomposition analysis (CDA) was run using the “optimize” function implemented in Ledalab (Benedek and Kaernbach 2010). From the CDA we extracted the global mean (the mean EDA value within the analyzed window, µS), the number, and the mean amplitude of EDA peaks. ECG and EDA were recorded during a 5 min resting state at the beginning of the experimental procedure, as well throughout the PASAT/PANRAT (1 min baseline + 3 min task).

### Data reduction

For the spontaneous tempo task, we considered the mean inter-response interval (IRI) as a measure of mean performance, and the coefficient of variation (CV) was used as a measure of variability (CV = SD/IRI).

To control for the “practice” trial that all participants had to perform before the PASAT/PANRAT, and therefore to account for baseline differences, we computed the HR and SCL percentage changes (i.e., the difference between HR and SCL during each of the three 1 min of the task minus the HR/SCL of the 1 min baseline divided by the baseline score).

For the time bisection task, a 7-point psychometric function was traced, plotting the seven comparison intervals on the x-axis and the probability of responding “long” on the y-axis. The cumulative normal function was fitted to the resulting curves. More specifically, we used a non-linear least squares analysis, with a Levenberg–Marquardt algorithm. We estimated the Bisection Point (BP, ms) defined as the *x-*value corresponding to the 0.50 probability of “long” responses on the *y-*axis, and BP served as an index of perceived duration: smaller the BP value indicates temporal overestimation (Kopec and Brody 2010). To obtain measures of accuracy, we compute the Constant Error (CE, ms) as the absolute difference between the BP and the midpoint (600 ms). Higher CE means less accurate performance (see Cellini et al. 2015 for a similar procedure). Moreover, as an indicator of temporal sensitivity, we estimate the Weber ratio (WR), which is based on the just noticeable difference (JND, i.e., the smallest change in the stimulus that produces a behavior change) divided by the BP. The JND was defined as the difference between estimated durations yielding 75% and 25% accuracy (Grondin 2008). For WR smaller value indicates higher temporal sensitivity.

### Statistical analyses

The Shapiro–Wilk test was used to test whether data were normally distributed. Independent *t* tests were conducted to compare responses to questionnaires, the performance in the spontaneous tempo task, and the subjective evaluation of valence and arousal after the PASAT and PANRAT tasks for data normally distributed, otherwise, we used the Mann–Whitney *U* test.

Changes in HR and EDA parameters recorded during the stress (PASAT) and control (PANRAT) conditions were included in a repeated measure ANOVA (rmANOVA) with *Group* (CONTROL vs. STRESS) as a between-subjects factor and *Time* (1, 2, and 3 min) as a within-subjects factor.

From the bisection task, we analyzed the CE and WR using a rmANOVA with *Group* (CONTROL vs. STRESS) as a between-subjects factor and *Block* (1, 2, 3, and 4) as a within-subjects factor. We also provided the mean BP values for better comparison with other studies.

For all the ANOVAs, in case of significant effect or interaction, the Holm test was used for post-hoc analysis. For all analyses, the significance level was set at *p* < 0.05, and Cohen’s *d*, the rank-biserial correlation, and *η*^2^_p_ values are reported as measures of effect size (ES).

## Results

Table 1 reports a summary of demographics and the self-report questionnaires. The two groups did not differ for any demographic, self-report variables, or all of the physiological indices besides the EDA global mean, which was higher in the CONTROL group compared to the STRESS group.

### Baseline time processing: spontaneous tempo task

**Fig. 2:**
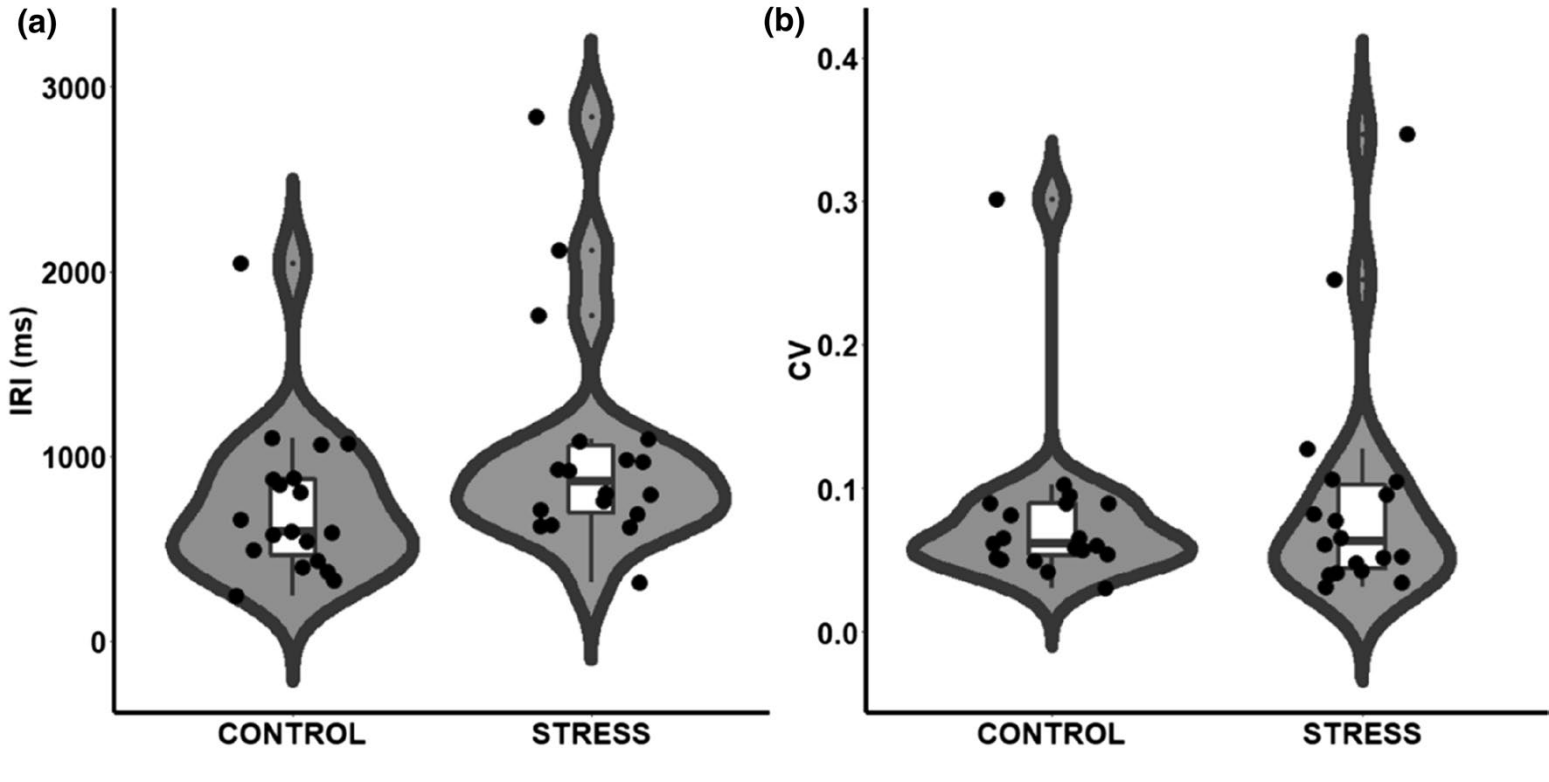
**a** Inter-trial intervals (IRI) and **b** coefficient of variation (CV) of the spontaneous tempo task as a function of Group (Control vs. Stress). Each dot represents a single participant

Since both IRI and CV were not normally distributed, we tested group differences using the Mann–Whitney *U* test. No significant differences in the spontaneous tempo performance were shown between the two groups (Fig. 2). However, a significant difference in the mean IRI was observed between the groups (Table 2).

**Table 2:** Summary of results of the spontaneous tempo task for the two groups

	Control		Stress		*U*	*p*	ES
	Mean	SD	Mean	SD			
IRI (ms)	1034.3	615.0	731.5	409.8	106.00	0.049	0.380
CV	0.09	0.08	0.08	0.06	152.50	0.576	0.108

*IRI* mean inter-response interval, *CV* coefficient of variation

To control for this result, we plotted the distribution of the responses (Fig. 2), which showed that this nominal difference was driven by a few outliers. We then recheck the data removing 4 outliers (data exceeding 3SD from the mean value of the sample). The Mann–Whitney *U* test for IRI became not significant (*U*(31) = 90.00, *p* = 0.108, *ES* = 0.333).

### Manipulation check

#### Physiological stress

**Fig. 3:**
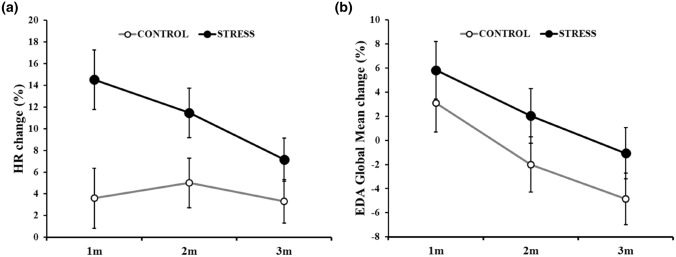
**a** HR and **b** EDA global mean change as a function of time during the auditory-paced tasks (PASAT for the STRESS group and PANRAT for the CONTROL group). Error bars represent standard errors of the mean

The rmANOVA on the HR showed a significant *Group* main effect [*F*(1,36) = 5.87, *p* = 0.021, *η*^2^_p_ = 0.140], with greater HR change in the STRESS group, and a *Time* main effect [*F*(2,72) = 4.06, *p* = 0.021, *η*^2^_p_ = 0.10] with HR accelerations that decreased from the first to the last minute of the task (1 m vs. 3 m: *pholm* = 0.057). We also observed a *Group* × *Time* interaction [*F*(2,72) = 3.21, *p* = 0.046, *η*^2^_p_ = 0.08; Fig. 3a], with the STRESS group showing nominal greater HR changes in the first minute compared to CONTROL (*pholm* = 0.097). Also, only the STRESS group showed a significant HR deceleration from the first to the last minute of the task (1 m vs. 3 m: *pholm* = 0.048).

The rmANOVA on the EDA global mean showed a significant *Time* main effect [*F*(2,72) = 25.84, *p* < 0.001, *η*^2^_p_ = 0.42), with a reduction of EDA across time (*pholm* < 0.001 for the comparison of each time point), but neither significant difference between groups [*F*(1,36) = 1.40, *p* = 0.244, *η*^2^_p_ = 0.04] or interaction [*F*(2,72) = 0.22, *p* = 0.806, *η*^2^_p_ < 0.01, Fig. 3b] were found.

The rmANOVA on the number of SCR showed no significant *Time* main effect [*F*(2,72) = 1.60, *p* < 0.209, *η*^2^_p_ = 0.04), and neither significant difference between groups [*F*(1,36) = 0.61, *p* = 0.439, *η*^2^_p_ = 0.02] or interaction [*F*(2,72) = 0.12, *p* = 0.887, *η*^2^_p_ < 0.01 were found.

The analysis of the SCR amplitude showed a significant *Time* main effect [*F*(2,72) = 26.952, *p* < 0.001, *η*^2^_p_ = 0.43), with a decrease in SCR amplitude between the first and the second minute (*pholm* < 0.001) but not between the last two minutes (*pholm* = 0.877). Again, neither the difference between groups [*F*(1,36) = 0.07, *p* = 0.787, *η*^2^_p_ < 0.01] or interaction [*F*(2,72) = 1.00, *p* = 0.372, *η*^2^_p_ = 0.03 were significant.

#### Subjective stress

**Fig. 4:**
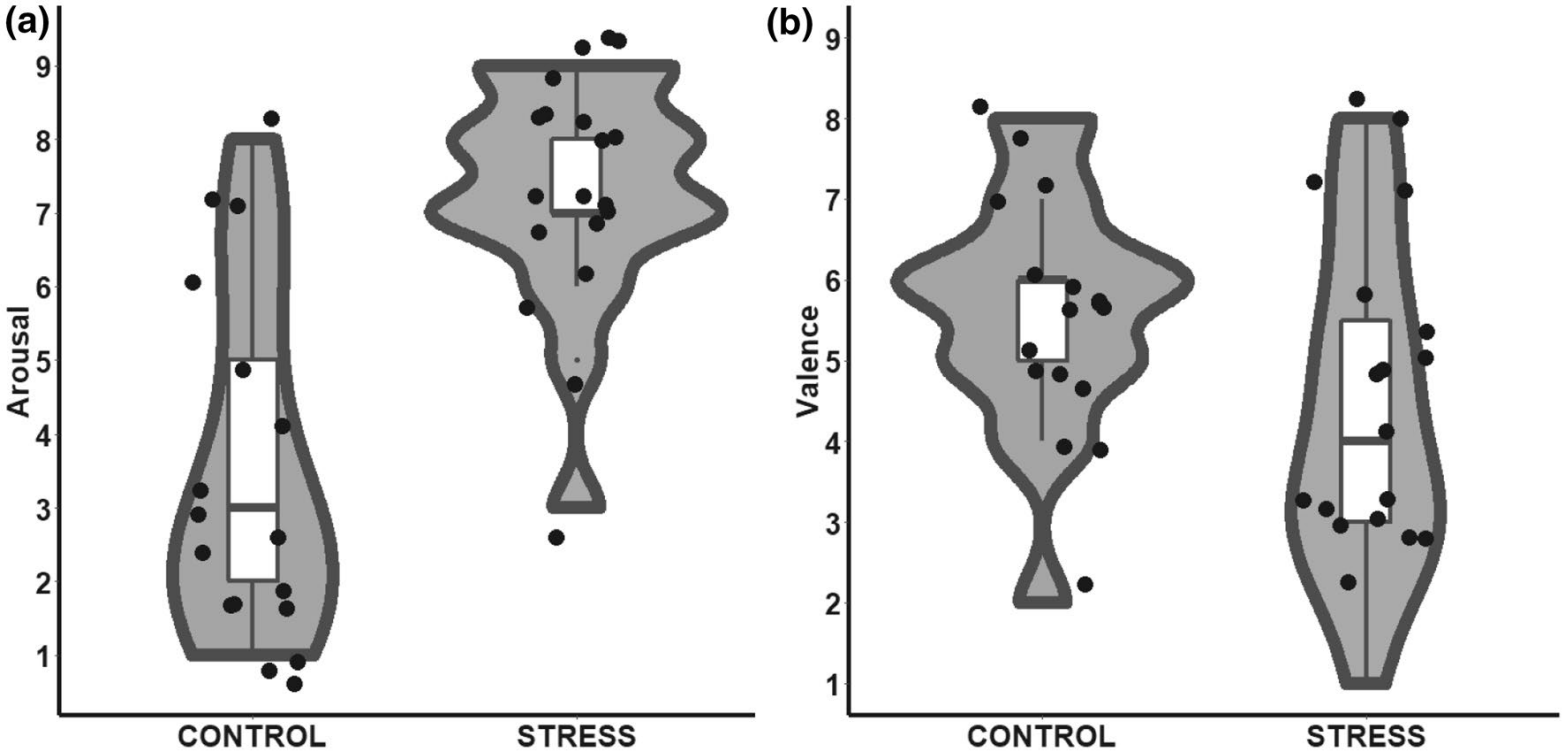
**a** Arousal and **b** Valence ratings of the auditory paced tasks (PASAT for the STRESS group and PANRAT for the CONTROL group). Each dot represents a single participant

At the end of the PASAT and PANRAT we asked our participant to rate the level of perceived stress in terms of valence (i.e., state of pleasantness evoked by performing the task) and arousal (i.e., state of activation evoked by performing the task). The PASAT (performed by the STRESS group) was rated as more arousing [*t*(34) = − 5.90, *p* < 0.001, Cohen’s *d* = − 1.97; Fig. 4a)] and less pleasant [*t*(34) = 2.04, *p* = 0.050, Cohen’s *d* = 0.68; Fig. 4b)] compared to the PANRAT (performed by the CONTROL group), indicating that the task was able to induce stress at a subjective level.

### Post-manipulation time perception: time bisection task

**Fig. 5:**
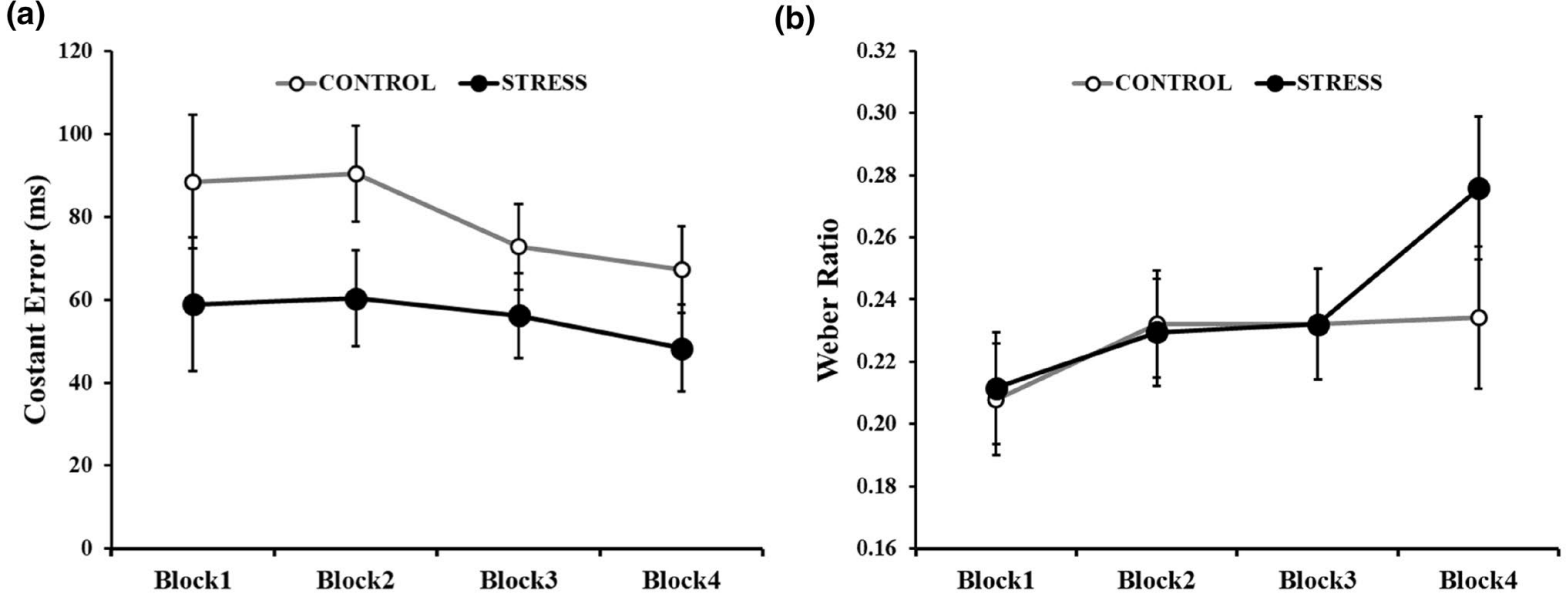
**a** Constant error (CE) and **b** Weber ratio (WR) as a function of Block and Group. The error bars represent the standard errors of the means

The rmANOVA conducted on CE revealed a main effect of *Group* [*F*(1, 36) = 5.05, *p* = 0.031, *η*^2^_p_ = 0.12 indicating higher accuracy in the STRESS group (mean CE = 31.05 ± 23.64) compared to the CONTROL group (mean CE = 65.95 ± 41.71, see Table 3). No main effect of *Block* [*F*(3, 108) = 1.05, *p* = 0.372, *η*^2^_p_ = 0.03] or interaction [*F*(3, 108) = 0.192, *p* = 0.902, *η*^2^_p_ = 0.01; see Fig. 5a] was found. Although the interaction was not significant, it can be seen that while the STRESS group maintained a low CE in all four blocks, the controls had a higher CE in the first two blocks, which decreased in the last two blocks, reaching a similar level as the STRESS group. These results indicate that after the stressing task participants were more accurate as their responses tended to be closer to the midpoint (i.e., 600 ms).

**Table 3:** Summary of main parameters of the time bisection task for the two groups across the four blocks

		Block 1	Block 2	Block 3	Block 4
CE (ms)	STRESS	58.9 ± 36.9	60.3 ± 50.7	56.2 ± 29.7	48.3 ± 42.6
	CONTROL	88.5 ± 92.3	90.3 ± 50.2	72.8 ± 56.1	67.3 ± 48.6
WR	STRESS	0.21 ± 0.08	0.23 ± 0.08	0.23 ± 0.08	0.28 ± 0.12
	CONTROL	0.21 ± 0.08	0.23 ± 0.07	0.23 ± 0.08	0.23 ± 0.08
BP (ms)	STRESS	612.92 ± 69.92	616.11 ± 78.65	593.77 ± 65.40	562.92 ± 57.39
	CONTROL	625.49 ± 93.74	641.63 ± 86.38	637.70 ± 85.58	582.15 ± 83.95

*CE* Constant error, *WR* Weber ratio, *BP* Bisection point

The rmANOVA conducted on WR revealed no main effects of *Group* or interaction and a trend for *Block* [*F*(3, 108) = 2.60, *p* = 0.056, *η*^2^_p_ = 0.07], with a nominal increase of WR across the blocks (Block 1 = 0.21, Block 2 = 0.23, Block 3 = 0.23 and Block 4 = 0.25, see Fig. 5b).

### Exploratory correlations

First, we explored whether individual differences (i.e., circadian preferences, sleepiness, and personality traits) were associated with spontaneous tempo performance, which was performed before any psychophysiological manipulation. We observed a negative association between the Conscientiousness trait and CV (Spearman *Rho* = − 0.354, *p* = 0.034) suggesting that participants more conscientious had a lower temporal variability.

Then, we conducted exploratory Pearson correlations between physiological stress**,** as indexed by the overall HR and EDA global mean changes during the PASAT/PANRAT in the whole sample, and the CE and WR averaged across the four blocks. All the correlations were not significant (all *r*’s <|0*.*265|, all *p*’s > 0.109).

We also conducted exploratory Spearman correlations between subjective arousal and valence (since these variables have ordinal data) and physiological arousal levels (overall HR and EDA global mean changes during the PASAT/PANRAT) in the whole sample. We found no significant associations between these variables (HR vs Valence: *Rho* = − 0.196, *p* = 0.253; HR vs Arousal: *Rho* = 0.319, *p* = 0.058; EDA vs Valence: *Rho* = − 0.027, *p* = 0.876; EDA vs Arousal: *Rho* = − 0.002, *p* = 0.989).

## Discussion

The present study aimed to examine whether psychophysiological stress can affect time perception in healthy participants. It is well known that variation in the arousal level can influence the subjective temporal perception of time by speeding up or slowing down the pace of the internal clock (Buhusi and Meck 2002, 2005; Droit-Volet and Meck 2007). To this end, participants in the experimental group were asked to perform a finger-tapping task and a time bisection task, before and after the stressing task, respectively, as measures of time perception. Moreover, we collected subjective ratings and data from two physiological indices, HR and EDA, to evaluate whether our stressful task would induce a stronger psychological and physiological reactivity compared to a control task. Notwithstanding a similar performance between the two groups at the spontaneous motor task, performed before any physiological manipulation, we observed an improved performance (i.e., higher accuracy) in the temporal bisection task in the participants who performed the stressful task compared to the controls.

In detail, participants in the two groups, which reported similar psychological traits and state characteristics, showed a similar baseline temporal performance at the spontaneous motor task before any stress manipulation. Note that this task has often been used as an index of the speed of the internal clock as well as to assess the integrity of internal timing mechanisms in a variety of neurological and neuropsychiatric disorders (Jones and Jahanshahi 2014; Mioni et al. 2016c). Importantly, as we showed before (Mioni et al. 2016b), the spontaneous tempo provides a reliable measure of subjective tempo.

Then, we successfully manipulated psychophysiological stress, as shown by the increased cardiac response and perceived arousal, and decrease perceived valence, in the PASAT (stressful task) compared to the PANRAT (non-stressful task). However, the EDA did not differ during the two tasks. This may be the result of the strong respiration pattern induced by both tasks, which has been shown to reliably induce skin responses, which tend to decrement their amplitude with repetition, (Kira et al. 2001; Seto-Poon et al. 2005). Note that the two tasks were identical in the duration, type of stimuli, and frequency of verbal responses.

Lastly, we showed that being in an activated state may indeed be helpful to process a very short duration (< 1 s), as highlighted by the lower constant error in the temporal bisection for the STESS group compared to the CONTROL group.

The higher accuracy in the more psychophysiologically aroused participants may seem counterintuitive since it can be expected that an arousing situation may bias the perception of an event, which usually is perceived as lasting longer (i.e., temporal overestimation; Droit-Volet and Meck 2007; Gil and Droit-Volet 2011; Lake et al. 2016). However, it should be remarked that in the current study, we induced both a psychological and physiological activation *before* the temporal task and not during timing estimation. We wanted to assess whether and how an already psychophysiologically-activated individual performs a temporal task. This idea is in line with some recent studies in which the individuals’ physiological activity was modulated before a timing task. For example, Ogden et al. (2019) manipulated both the valence of the to-be-time stimuli (unpleasant and neutral pictures) and the participants’ pre-task parasympathetic activity using normal and paced-breathing exercises. They showed that irrespective of picture valence, pre-task lower parasympathetic activity was associated with higher temporal accuracy in a verbal temporal estimation of duration < 1 s. Moreover, a study using a social stressor before and after a temporal reproduction showed a post-stress lengthening in the reproduction of pleasant and unpleasant pictures compared to pre-stress manipulation, with no change for the neutral ones (van Hedger et al. 2017). The main difference between these studies and ours is that we did not use arousing pictures as stimuli to-be-timed. Indeed, our study design is more similar to Dormal et al. (2018), who modulated physical activity using cycling (high activation), crosswords (low activation), and relaxation (very low activation), before a verbal temporal categorization task of duration below 1 s. They showed oral over-estimation after HR manipulation irrespective of the specific manipulation. However, compared to Dormal et al. (2018), we induced both psychological and physiological arousal before the timing task and we assessed temporal performance using psychometric functions. Therefore, our results are difficult to compare to previous literature. Nevertheless, all these studies, including ours, are showing that physiological changes before a timing task indeed affect temporal processing. However, further studies are needed to clarify how physiological activity modulates temporal processing.

Interestingly, looking at the accuracy in the temporal bisection task across the blocks, we observed that the controls improved their accuracy (although not in a statistically significant way) in the last two blocks compared to the first two, suggesting a learning effect through the task. This learning effect can be explained by taking into account the idea that subjective perception of time, as assessed with tasks such as time discrimination, bisection, or reproduction, may rely on a relative, rather than absolute, coding scale, in particular, if multiple intervals are randomly presented within each block (as it was done in our study). In this sense, even if we presented the two standard intervals, participants form a memory representation of “time” that is updated trial-by-trial (Jones and McAuley 2005; Mioni et al. 2014). This is consistent with what is revealed by Vierordt’s law (Lejeune and Wearden 2009): when short and long intervals are presented within the same experimental context, shorter intervals tend to be overestimated and longer intervals are underestimated. Jones and McAuley (2005) tried to give a comprehensive explanation of temporal performance using time discrimination tasks. They reported interesting results describing local and global context effects. The authors pointed out that the temporal context systematically affects the perception of a temporal interval. Mioni et al. 2014 further explored this issue indicating that the subjective estimation of 1 s is influenced by the range of the intervals presented within the same experimental session. In the present study, we interpreted the reduction of CE in the control group in terms of learning effect suggesting that participants going through the task updated the memory representation of time, decreasing the CE as an index of increased accuracy. On the contrary, participants in the STRESS condition already formed a more accurate representation of time that was maintained stable through the four blocks.

This result induces us to speculate that an increase in psychophysiological arousal before a temporal task may help to optimize the cognitive resources required to adequately perform the task. Indeed, the temporal bisection task, like most cognitive tasks, can be considered a mild “stressor”. As reported by several studies, when facing a cognitive challenge, we need to mobilize cognitive and physiological resources to perform appropriately (Silvestrini and Gendolla 2019; Westbrook and Braver 2015). In the current study, the STRESS group started the temporal task with an increased psychophysiological activation (both cardiac and subjective), which may have helped them to quickly adapt to the task demands.

Another explanation refers to the internal clock models, which proposes that the pacemaker rate is modulated by arousal, with a speeding-up of the internal clock system in case of a higher arousal level (Buhusi and Meck 2002, 2005; Droit-Volet and Meck 2007). We speculate here that psychological and physiological arousal before a timing task can increase the “sampling rate” of the clock system, allowing the collection and storage of more information. When this information is sent to the decision stage of the SET model, the system can be more accurate in comparing the temporal information of different stimuli.

In the current study, we also collected information about personality traits and circadian preferences, variables that have been previously associated with time perspective (e.g., Bisson and Grondin 2020; Hammerschmidt and Wöllner 2023; Hornik et al. 2010; Momi et al. 2023; Rammsayer 1997). We did not observe any trait differences across the groups, nor any associations between spontaneous tempo and circadian preferences, state anxiety, and perceived sleepiness. However, we observed negative associations between conscientiousness and CV in the whole sample, suggesting that participants more conscientious were the ones with better temporal sensitivity (higher precision in their spontaneous tapping).

The results of the current study should be interpreted taking into account some limitations. First, although the sample size was designed considering a medium effect size for the main comparison (i.e., change in CE across the four blocks in the two conditions), it is possible that a larger sample would be more appropriate to identify smaller effects. Second, based on previous literature that the physiological activation due to the PASAT would be maintained in the subsequent task (Starcke et al. 2016; Bachmann et al. 2019). However, we cannot exclude that the difference in psychophysiological arousal dissipated in the following minutes (see Tanosoto et al. 2012) or the participants in the control task reached a level of activation similar to the stress group during the time bisection task. Therefore, further study may capitalize on reliable stressful tasks which effect are known to last for several minutes.

To conclude, here we showed that psychological and physiological stress can alter subsequent temporal accuracy. Future studies should aim at replicating the current findings with similar and different temporal tasks, including different temporal ranges (e.g., seconds). Moreover, it is important to design studies aimed at understanding whether the observed improvement in temporal processing depends on an extra mobilization of physiological and cognitive resources or a modulation of the sampling rate of temporal information.

## Data Availability

The data that support the findings of this study are available from the corresponding author upon reasonable request. The study was not preregistered. No custom code was made to analyze the data.
